# Manipulating expectancies in optometry practice: Ocular accommodation and stereoacuity are sensitive to placebo and nocebo effects

**DOI:** 10.1111/opo.13036

**Published:** 2022-08-12

**Authors:** Jesús Vera, Beatriz Redondo, Elena Ocaso, Sara Martinez‐Guillorme, Rubén Molina, Raimundo Jiménez

**Affiliations:** ^1^ CLARO (Clinical and Laboratory Applications of Research in Optometry) Research Group, Department of Optics, Faculty of Sciences University of Granada Granada Spain; ^2^ Óptica del Penedes Optometry Center Zaragoza Spain; ^3^ Garcia Ópticos Optometry Center Cintruenigo Spain

**Keywords:** accommodative response, depth perception, nocebo, placebo, variability of accommodation, visual examination

## Abstract

**Introduction:**

There is scientific evidence that an individual's beliefs and/or expectations play a role in the behavioural and physiological response to a given treatment. This study aimed to assess whether the dynamics of the accommodative response and stereoacuity are sensitive to experimentally induced placebo and nocebo effects.

**Methods:**

Nineteen healthy university students performed three experimental sessions (placebo, nocebo and control) in randomised order, with the dynamics of the accommodative response (magnitude and variability), stereoacuity and subjective measures being assessed in all sessions. For the experimental manipulation, participants ingested an inert capsule that was alleged to have positive (white capsule, placebo condition) or negative (yellow capsule, nocebo conditions) effects on the human physiology. In the control condition, participants did not ingest a capsule.

**Results:**

The data revealed that the variability of accommodation was sensitive to experimentally induced placebo and nocebo effects, showing a more stable accommodative response for the placebo compared with the nocebo condition (corrected *p*‐value = 0.04, Cohen's *d* = 0.60). In addition, better stereoacuity was found with the placebo, compared with the nocebo (corrected *p*‐value = 0.01, Cohen's *d* = 0.69) and control (corrected *p*‐value = 0.03, Cohen's *d* = 0.59) conditions. Successful experimental manipulation was confirmed by the analysis of subjective perceptions.

**Conclusions:**

These findings provide evidence that manipulating expectations about the efficacy of an inert treatment affect the dynamics of the accommodative response (variability of accommodation) and stereoacuity. The results have important applications in both clinical and research outcomes, where individuals´ beliefs/expectations could modulate the visual function.

**Key points**

- Factors associated with the treatment context and patients' beliefs/expectations modulate the physiological response to a given treatment.
- The results of this study show that placebo/nocebo manipulation has an effect on the stability of the accommodative response and stereoacuity.
- Manipulating expectations about the efficacy of an inert treatment alters visual functioning, which should be considered by clinicians and researchers.

## INTRODUCTION

Within the field of health sciences, research efforts are focused on determining the most effective medical and/or psychosocial strategies for preventing and managing health conditions. In addition to the specific effects of the treatment itself, there are other factors associated with the treatment context and patients' beliefs and/or expectations that may positively (placebo) or negatively (nocebo) affect the response to treatment.^1^ There are claims that placebo and medical treatment effects can work synergistically to manage health conditions better, suggesting that clinicians should consider these mechanisms in their daily routine.^2^, ^3^

Motivation and expectancy play an important role in perceptual encoding and recognition since they direct attention to the stimulus for further processing.^4^ Scientific evidence suggests that several indices of the ocular dynamics (e.g., pupil size, accommodative response or eye movements) share neural mechanisms with the attention‐regulating centres.^5^, ^6^, ^7^, ^8^, ^9^ Indeed, manipulation of the attentional state has been linked with changes in the dynamics of several visual indices such as the accommodative response,^10^, ^11^, ^12^, ^13^ pupil size^7^, ^14^ or eye movements.^8^, ^15^ Specifically, with regard to the dynamics of the accommodative response, Redondo and colleagues^11^ found that the use of attentional facilitators (auditory biofeedback) and distractors (dual‐tasking) influenced both the lag and variability of accommodation. Molina et al.^13^ observed that capturing attention by the use of engaging visual stimuli caused a heightened accommodative response in children with attention deficit hyperactivity disorder.

It is well‐known that placebo and nocebo effects are derived from highly active processes in the brain that are mediated by psychological mechanisms such as expectation and conditioning.^16^ These effects can represent both strength and vulnerability in the cardiovascular and brain response to a diagnostic examination or types of therapy,^17^, ^18^, ^19^, ^20^, ^21^, ^22^ and have been demonstrated to modulate visual attention, alter visual search behaviour and induce activation in brain areas involved in visual perception.^23^, ^24^, ^25^ To the best of our knowledge, there are only two studies that have investigated the visual function response to placebo or nocebo effects, but they were focused on oculomotor function.^26^, ^27^ Therefore, given that placebo and nocebo effects can induce changes in measurements within both subjective and objective domains,^28^ it is of interest to evaluate the influence of placebo and nocebo effects on measures of the visual function such as the dynamics of the accommodative response and stereoacuity.

In health sciences, and specifically in the fields of optometry and vision sciences, understanding the placebo and nocebo phenomenon might have important implications in everyday clinical practice because it could constitute a new strategy for the management of visual dysfunctions (such as in visual therapy). It is of special relevance in research settings due to the importance of controlling these effects when designing a research study or clinical trial.^1^ Based on the research gap in relation to placebo and nocebo effects on the visual function, the main objectives of this study were: (1) to examine the influence of placebo and nocebo effects on the dynamics of the accommodative response and (2) to assess whether stereoacuity is sensitive to placebo and nocebo effects. In addition, we determined subjective perceptions of activation 30 min after intake of the placebo/nocebo. Based on the accumulated scientific evidence, we hypothesise that manipulation of participants' expectations will influence ocular accommodation and stereoacuity, as has been shown for several other physiological measures.^17^, ^18^, ^19^, ^20^, ^21^

## METHODS

### Participants and ethics approval

Nineteen healthy university students studying for different academic degrees (12 women; age = 22.6 ± 3.5 years) took part in this study. All participants were screened by a board‐certified optometrist based on the following inclusion criteria: corrected visual acuity in both eyes of 0.0 log MAR or better; no anisometropia or astigmatism >1.50 D; low levels of visual discomfort (<24) as measured with the Conlon survey (average score = 10.8 ± 6.4)^29^; no accommodative and binocular dysfunction based on the recommendations of Scheiman and Wick^30^ or a history of treatment for such a dysfunction; no history of refractive surgery, orthokeratology, strabismus or amblyopia; and no current systemic disease or pharmacological treatment. Participants were instructed to abstain from alcohol and caffeinated drinks for 24 and 12 h, respectively, and to sleep for at least 7 h during the night before each experimental session.^31^, ^32^, ^33^, ^34^, ^35^ The study followed the tenets of the Declaration of Helsinki and was approved by the University of Granada Institutional Review Board (IRB approval: 438/CEIH/2017). All participants gave informed consent before enrolment in this investigation.

### Experimental design

The study was designed to examine the acute effects of placebo/nocebo on the dynamics of ocular accommodation (lag and variability of accommodation) at three viewing distances as well as stereoacuity. The within factors were the manipulation of participants' beliefs after ingesting a capsule (placebo, nocebo and control) and viewing distance (500, 40 and 20 cm). The dependent variables were accommodative lag, accommodative variability, stereoacuity and subjective measures.

### Dependent variables

#### Dynamics of the accommodative response

For assessment of the magnitude and variability of the accommodative response, we used the clinically validated binocular open‐field Grand Seiko WAM‐5500 autorefractor (Grand Seiko, grandseiko.com) in HI‐SPEED mode under binocular conditions.^36^ This instrument permits a dynamic recording of ocular refraction at a rate of ~5 Hz, with a sensitivity of 0.01 D. First, we obtained a baseline static measure of refractive error in both corrected eyes while participants viewed a 5 m stationary target, and these measurements were used to calculate the lag of accommodation. Then, the accommodative response was measured dynamically under binocular viewing conditions. Data were gathered from the dominant eye, as determined by the hole‐in‐the‐card method.^37^ Measurements were obtained for three viewing distances (500, 40 and 20 cm) with 60 s of recording at each distance. This duration has been proposed as the optimal recording time for measuring the steady‐state accommodative response.^38^ Participants were seated at the instrument with their head stabilised in the chin rest and forehead strap and were aligned with the fixation target positioned in midline gaze. The fixation target comprised a black high‐contrast (Michelson contrast = 79%) Maltese cross on a white background. The same target was used for the three viewing distances to create viewing angles of 2.29°, 2.86° and 5.73°, respectively. Laboratory illumination was kept constant at ~150 lux during the experiment (Illuminance Meter T‐10A, Konica Minolta, konicaminolta.eu). Participants were instructed to maintain the stimulus as clear as possible during the 60‐s period.

In order to discard blinking or recording errors, we removed data points ± 3 standard deviations away from the mean spherical refraction value.^39^, ^40^ The lag of accommodation was calculated using the equation proposed by Poltavski et al.,^10^ by subtracting the mean value obtained dynamically from the accommodative demand at each distance after correction for any residual refractive error (the latter being determined statically while fixating a far target). The variability of the accommodative response was obtained from the standard deviation of the dynamic accommodation recordings.^41^

#### Stereoacuity

Stereoacuity for a real depth stimulus was measured using the Frisby stereo test (Haag‐Streit Clement Clarke; haag‐streit.com/clement‐clarke/), which allows assessment of depth perception with only binocular parallax cues. In order to minimise monocular parallax clues, we used a chin and forehead support to ensure that participants did not tilt or move their head during testing.^42^ A four‐alternative forced‐choice paradigm following a staircase procedure, as described by Costa and colleagues,^43^ was used to measure stereoacuity, with the subject having to indicate verbally which of the four squares contained a small circle which appeared to lie in a different plane. Following the manufacturer's instructions, an experienced examiner varied the plate thickness (6, 3 and 1 mm), target orientation and viewing distance in order to determine the smallest angle of disparity that could be discriminated by the subject.

#### Subjective measures

The Stanford Sleepiness Scale (SSS) was used to assess the levels of alertness/sleepiness at the beginning of each experimental session.^44^ The SSS is a self‐rating scale that includes seven statements ranging from 1 ‘Feeling active, vital, alert or wide awake’ to 7 ‘No longer fighting sleep, sleep onset soon, having dream‐like thoughts’. Participants were asked to report which statement described their actual state. After 30 min of ingesting the corresponding capsule (placebo, nocebo or control), participants used a visual analogue scale to report their perceived levels of activation. This scale ranges from 1 ‘absolutely not activated’ to 10 ‘extremely activated’.

### Procedure and placebo/nocebo manipulation

**FIGURE 1:**
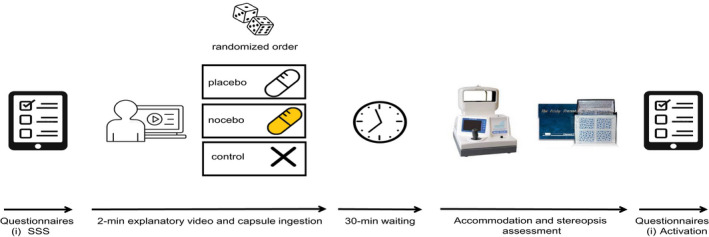
Schematic illustration of the procedure followed in the main experimental sessions (sessions 2, 3 and 4).

Participants visited the laboratory on four separate occasions on different days, and all sessions were scheduled at the same time of the day (±1 h) to avoid Circadian variations. The first session was used for screening the inclusion criteria and to obtain demographic data (see the Participants and Ethical Approval subsection). The second, third and fourth visits (main experimental sessions) were identical, with the exception of the instructions given to the participants. Figure 1 depicts a schematic illustration of the procedure followed in the main sessions. For the experimental manipulation, participants ingested an inert capsule with instructions indicating that it would either enhance (placebo) or reduce (nocebo) visual performance. Upon arrival at the laboratory, participants first completed the SSS. Then, participants watched a 2‐min video, which described the ‘alleged positive (placebo) or negative (nocebo) effects’ of the capsule that they were about to ingest. In the placebo video, a researcher explained the ‘alleged positive effects’ of the white capsule, indicating that this included substances known to enhance the level of alertness and has been demonstrated to improve cognitive and physical performance. In the nocebo condition, a researcher explained the ‘alleged negative effects’ of the yellow capsule that they were about to ingest, indicating that this capsule was composed of substances known to reduce the level of alertness and cause drowsiness, with this composition of substances being associated with worse cognitive and physical performance. In the control video, a researcher explained that they did not have to ingest any substance because this session will be used as a control condition to compare with the other two experimental trials. The order of the three experimental conditions was randomised across subjects. Both capsules (placebo and nocebo) were empty. Opaque gelatine capsules were employed for the placebo and nocebo conditions, using a white and a yellow capsule for the placebo and nocebo trials, respectively. No capsule was ingested in the control condition (see Figure 1). After taking the capsule, participants rested for 30 min, and then, ocular accommodation and stereoacuity were assessed as described above (see the Dependent variables subsection). Lastly, participants reported their perceived level of activation using a visual analogue scale. The examiner was blinded to the experimental condition, with the corresponding capsule and explanatory video being provided by a third person.

### Statistical analyses

Before analysis, we checked the normality of the data (Shapiro–Wilk test) and the homogeneity of variances (Levene's test) (*p* > 0.05 in all cases). Subsequently, two repeated measures analyses of variance (ANOVAs), with ‘experimental manipulation’ (placebo, nocebo and control) and ‘target distance’ (500, 40 and 20 cm) as within‐participants factors, were performed for the lag and variability of the accommodative response. To determine the placebo and nocebo effects on stereoacuity and perceived levels of activation, we conducted an unifactorial ANOVA with the ‘experimental manipulation’ (placebo, nocebo and control) as the only within‐participant factor. The magnitude of the differences was reported as partial eta squared (*ƞp* ^2^) and Cohen's effect size (ES) for Fs and t‐tests, respectively. Statistical significance was set at an alpha level of 0.05, and *post‐hoc* tests were corrected with the Holm‐Bonferroni procedure.

## RESULTS

Table 1 shows descriptive values for all the subjective and objective visual measures assessed in this study.

**TABLE 1:** Descriptive (mean ± standard deviation) values for the dependent variables of this study

	Placebo	Nocebo	Control
Subjective perceptions			
Stanford Sleepiness Scale (1–7)	1.95 ± 0.97	1.84 ± 0.76	1.89 ± 0.81
Level of activation (1–10)	8.16 ± 0.83	6.79 ± 1.40	7.68 ± 1.20
Lag of accommodation (D)			
500 cm	0.18 ± 0.09	0.27 ± 0.18	0.26 ± 0.28
40 cm	0.88 ± 0.58	0.90 ± 0.58	0.93 ± 0.58
20 cm	1.08 ± 0.73	1.06 ± 0.67	1.10 ± 0.71
Variability of accommodation (D)			
500 cm	0.16 ± 0.07	0.17 ± 0.06	0.19 ± 0.10
40 cm	0.42 ± 0.14	0.52 ± 0.17	0.47 ± 0.19
20 cm	0.83 ± 0.30	1.02 ± 0.39	0.94 ± 0.41
Stereopsis (seconds of arc)	11.05 ± 7.18	17.89 ± 8.39	16.84 ± 9.46

### Effectiveness of the experimental manipulation

Participants reported similar levels of sleepiness/alertness at the beginning of each of the three experimental sessions, as shown by the analysis of the SSS (*F*_2,36_ = 0.1, *p* = 0.90). For the level of activation, we checked whether the manipulation resulted primarily in a placebo/nocebo effect in the three experimental conditions. We found a statistically significant effect for ‘experimental manipulation’ (*F*_2,36_ = 11.4, *p* < 0.001, η^2^ = 0.39); obtaining higher levels of perceived activation in the placebo when compared to the nocebo (corrected *p*‐value < 0.001, Cohen's *d* = 1.08) and control (corrected *p*‐value = 0.01, Cohen's *d* = 0.71) conditions. However, no statistically significant differences were observed for the comparison between nocebo and control (corrected *p*‐value = 0.34, Cohen's *d* = 0.37).

### Placebo and nocebo effects on the lag and variability of accommodation

**FIGURE 2:**
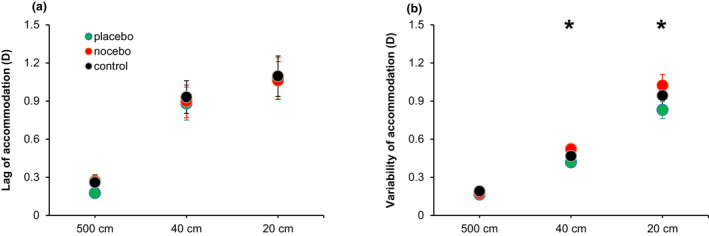
Effects of placebo/nocebo manipulation on the lag (panel a) and variability (panel b) of accommodation. *Denotes statistically significant differences between the placebo and nocebo conditions (corrected *p*‐value < 0.05). Error bars indicate the standard error, and all values are calculated across participants (*n* = 19).

Analysis of lag of accommodation showed a main effect of ‘target distance’ (*F*_2,36_ = 25.2, *p* < 0.001, η^2^ = 0.53), with lag of accommodation being negatively associated with the target distance (Figure 2, panel a). However, the main effect of ‘experimental manipulation’, nor the interaction ‘experimental manipulation × target distance’ did not reach statistical significance (*F*_2,36_ = 0.5, *p* = 0.60; and *F*_4,74_ = 1.3, *p* = 0.27; respectively).

For the variability of accommodation, the data revealed statistically significant differences for ‘experimental manipulation’ (*F*_2,36_ = 3.4, *p* = 0.04, η^2^ = 0.01) and ‘target distance’ (*F*_2,36_ = 122.1, *p* < 0.001, η^2^ = 0.73), but not for the interaction ‘experimental manipulation × target distance’ (*F*_4,74_ = 1.8, *p* = 0.15). *Post‐hoc* analyses revealed a lower variability of accommodation for the placebo than the nocebo condition (corrected *p*‐value = 0.04, Cohen's *d* = 0.60). With regard to the target distance, a more stable accommodative response (lower variability) was found for measurements taken at 500 cm compared with 40 cm (corrected *p*‐value < 0.001, Cohen's *d* = 1.38) and 20 cm (corrected *p*‐value < 0.001, Cohen's *d* = 3.56), as well as for 40 versus 20 cm (corrected *p*‐value < 0.001, Cohen's *d* = 2.18) (Figure 2, panel b).

### Placebo and nocebo effects on stereoacuity

**FIGURE 3:**
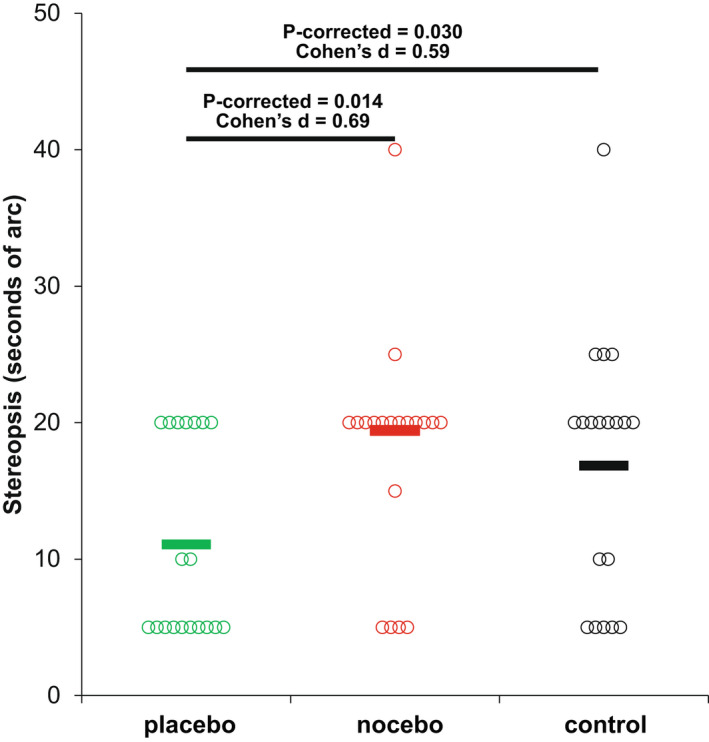
Scatterplot of the three experimental conditions on stereoacuity. The horizontal lines indicate the average value, and statistically significant differences (corrected *p*‐value < 0.05) with the corresponding effect size (Cohen's *d*) are depicted.

Stereoacuity was sensitive to the ‘experimental manipulation’ (*F*_2,36_ = 5.3, *p* = 0.01, η^2^ = 0.23), with participants achieving a greater stereoacuity in the placebo trial when compared to the nocebo (corrected *p*‐value = 0.01, Cohen's *d* = 0.69) and control (corrected *p*‐value = 0.03, Cohen's *d* = 0.59) conditions. However, the nocebo and control conditions were not significantly different from one another (corrected *p*‐value = 0.65, Cohen's *d* = 0.11) (Figure 3).

## DISCUSSION

The present study showed that variability of the accommodative response and stereoacuity were sensitive to experimentally induced expectations. Successful placebo/nocebo manipulation was confirmed by the perceived levels of activation reported by participants. Taken together, these findings provide evidence that both ocular accommodation and stereoacuity can be influenced by manipulating expectations and belief about the efficacy of an inert treatment. These results have important implications with regard to the development of strategies for the evaluation and management of various visual dysfunctions, as well as for the design of experimental studies in the field of vision sciences where placebo/nocebo effects may play a significant role. In addition, the data confirm that the lag and variability of the accommodative response are strongly dependent upon the accommodative demand, showing a greater lag and variability at closer viewing distances.^45^, ^46^

Using accordant verbal instructions is of great relevance for enhancing outcome expectations, and subsequently, placebo/nocebo efficacy.^21^ Analysis of subjective perceptions has been used routinely for examining the success of placebo/nocebo manipulation.^47^, ^48^, ^49^ For example, Mlynski et al.^17^ found that ingestion of an inert herbal supplement, which was described as having positive or negative effects on blood flow, modulated subjective perceptions of mental clarity. We explained to our participants that one capsule enhanced the level of alertness and improved behavioural performance (placebo), whereas the other capsule reduced the level of alertness and provoked drowsiness (nocebo). This manipulation produced an increase in perceived levels of activation for the positive instruction condition (placebo) and a reduction in levels of activation for the negative instruction condition (nocebo). Therefore, this subjective valuation report confirms the desired placebo/nocebo manipulation.

The placebo and nocebo effects on oculomotor function, as measured by eye‐tracking devices, have been investigated; however, the results derived from these studies were inconclusive.^26^, ^27^ In this regard, Schienle et al.^26^ showed that placebo manipulation increased the number of fixations for disgusting images displayed on a computer screen, whereas Höfler et al.^27^ found that nocebo suggestions might evoke improvements in visuospatial attention (more fixations) by inducing compensatory behaviour. Here, analysis of the variability of the accommodative response reveals that experimentally induced expectancy modulates this ocular index. Specifically, there was a more stable accommodative response associated with the placebo while viewing targets placed at 40 and 20 cm, with increased stability ranging between 10% and 20%, compared with the control and nocebo conditions.

These results agree with previous studies conducted by Redondo and colleagues,^11^, ^12^ who found that manipulating the level of engagement using attentional facilitators, such as auditory biofeedback or an engaging stimulus, produce a more stable accommodative response in both healthy subjects and children with attention deficit hyperactivity disorder at near distances. Similarly, Roberts et al.^50^ observed that accommodation stability is enhanced when using an engaging 10‐min visual task compared with passive viewing in children. Hynes et al.^51^ found a negative association between accommodative microfluctuations (i.e., the power of the high‐frequency component) and cognitive demand in an arithmetic task, which may suggest a similar effect to the nocebo manipulation and mentally demanding situations (i.e., dual tasking). Taken together, the recruitment of attentional resources by different means seems to have a positive effect on the variability of the accommodative response, as supported by the manipulation of expectations performed in the current study, which directed attention to the visual stimulus.

Contrary to the hypothesis formulated for the current experimental design, the magnitude of the accommodative response was insensitive to the placebo/nocebo manipulation. Related studies have obtained mixed results. For example, Wagner et al.^52^ found that accommodation training based on auditory biofeedback reduced the lag of accommodation, whereas Redondo et al.^12^ found that increasing attentional engagement by the use of different visual stimuli did not alter the accommodative lag in either a clinical or a control population. These differences may be attributable to variations in the type of manipulation, measurement method or individual differences. Indeed, the use of auditory biofeedback has been shown to impact the lag of accommodation, but only for targets placed at 20 cm and when the level of biofeedback was high (i.e., no effect for 500 and 40 cm viewing distance or for the low‐biofeedback condition).^11^ Further studies are required regarding this topic.

The assessment of stereoacuity requires subject co‐operation,^53^ and thus, the utility of this measurement may be limited in populations that are not sufficiently co‐operative (e.g., preschool children or persons with neurological disorders).^54^ As noted by Westheimer,^55^ depth perception depends on components such as visual input, as well as the individuals' attention, expectation, memory and learning. Due to the subjective nature of the stereoacuity tests used in optometric clinic settings, it is plausible that stereoacuity could be sensitive to placebo/nocebo manipulation. Data from the present study confirmed that stereoacuity is modulated as a function of performance expectations, and better values of stereoacuity are obtained after placebo ingestion in comparison with the nocebo and control conditions (average improvements of 6.8 and 5.8 s of arc, respectively). On this basis, eye care practitioners should consider the mediating role of non‐optical factors (expectancy, motivation and beliefs) for the assessment of depth perception in clinical and laboratory scenarios.

### Potential applications and limitations of the current study

Research in the fields of psychiatry and psychology has proven that factors such as clinical context, individual perception, imagination or expectancy play a role in the physiological response to a given treatment.^48^ In the present study, manipulation of performance expectations, using the verbal instructions given for an inert drug (empty capsule), modulated visual functioning (accommodative dynamics and stereoacuity) in a sample of healthy young adults. These findings may be useful in several ways: the manipulation of expectations could be used for the enhancement of treatment response or mitigation of side effects by eye care practitioners in their clinical practice, and the participation of different examiners or the lack of a double‐blind procedure in research could be a source of errors due to differences in instructions or behaviour. Therefore, these factors should be taken into consideration when appropriate.

This research incorporates novel insights into the field of vision sciences, showing that placebo/nocebo can affect visual function. However, the results of this investigation should be interpreted in the context of some limitations. Firstly, participants were university students studying for different academic degrees at the University of Granada who were free of any visual dysfunction, and thus, further studies are required to determine the generalisability of the current findings to broader populations with conditions such as accommodative insufficiency or infacility. Secondly, substantial individual differences have been reported for placebo responses, with personality factors playing an important role in this regard.^56^, ^57^ The mediating role of these factors should be explored in future studies. Thirdly, the characteristics of the fixation target (e.g., target size and luminance) or participants' refractive error are known to affect the dynamics of the accommodative response, and therefore, these findings should be interpreted with some caution.^45^, ^46^, ^58^, ^59^ Fourthly, the clinical relevance of the results is somewhat modest. Nevertheless, in a clinical situation where the instructions given to the patient are more explicit, it is reasonable to expect larger differences than those observed here. Future studies are needed in this regard. Lastly, the current results cannot be extrapolated to other visual measures or types of placebo/nocebo manipulation.

## CONCLUSIONS

The present study is the first to examine the effect of manipulating performance expectations on the dynamics of ocular accommodation and stereoacuity. In addition to subjective perceptions, we found a placebo response on the stability of the accommodative response (lower variability) and stereoacuity (better depth perception), with these effects being more evident when compared with the nocebo condition. However, the lag of accommodation was not modulated as consequence of placebo/nocebo manipulation. These results indicate that manipulating expectations regarding the efficacy of an inert treatment can influence visual functioning in the short term, which may be of relevance in both clinical and laboratory settings.

## AUTHOR CONTRIBUTIONS

**Jesus Vera:** Conceptualization (equal); data curation (equal); formal analysis (equal); methodology (equal); supervision (equal); visualization (equal); writing – original draft (equal). **Beatriz Redondo Cabrera:** Conceptualization (equal); data curation (equal); investigation (equal); visualization (equal); writing – review and editing (equal). **Elena Ocaso:** Data curation (equal); investigation (equal); methodology (equal); writing – review and editing (equal). **Sara Martinez‐Guillorme:** Data curation (equal); investigation (equal); methodology (equal); writing – review and editing (equal). **Rubén Molina Romero:** Data curation (equal); investigation (equal); writing – review and editing (equal). **Raimundo Jimenez:** Conceptualization (equal); project administration (equal); resources (equal); supervision (equal); writing – review and editing (equal).

## CONFLICT OF INTEREST

The authors report no conflicts of interest and have no proprietary interest in any of the materials mentioned in this article.
